# Predator Dispersal Determines the Effect of Connectivity on Prey Diversity

**DOI:** 10.1371/journal.pone.0029071

**Published:** 2011-12-16

**Authors:** Romana Limberger, Stephen A. Wickham

**Affiliations:** Department of Organismic Biology, University of Salzburg, Salzburg, Austria; University of Utah, United States of America

## Abstract

Linking local communities to a metacommunity can positively affect diversity by enabling immigration of dispersal-limited species and maintenance of sink populations. However, connectivity can also negatively affect diversity by allowing the spread of strong competitors or predators. In a microcosm experiment with five ciliate species as prey and a copepod as an efficient generalist predator, we analysed the effect of connectivity on prey species richness in metacommunities that were either unconnected, connected for the prey, or connected for both prey and predator. Presence and absence of predator dispersal was cross-classified with low and high connectivity. The effect of connectivity on local and regional richness strongly depended on whether corridors were open for the predator. Local richness was initially positively affected by connectivity through rescue of species from stochastic extinctions. With predator dispersal, however, this positive effect soon turned negative as the predator spread over the metacommunity. Regional richness was unaffected by connectivity when local communities were connected only for the prey, while predator dispersal resulted in a pronounced decrease of regional richness. The level of connectivity influenced the speed of richness decline, with regional species extinctions being delayed for one week in weakly connected metacommunities. While connectivity enabled rescue of prey species from stochastic extinctions, deterministic extinctions due to predation were not overcome through reimmigration from predator-free refuges. Prey reimmigrating into these sink habitats appeared to be directly converted into increased predator abundance. Connectivity thus had a positive effect on the predator, even when the predator was not dispersing itself. Our study illustrates that dispersal of a species with strong negative effects on other community members shapes the dispersal-diversity relationship. When connections enable the spread of a generalist predator, positive effects of connectivity on prey species richness are outweighed by regional extinctions through predation.

## Introduction

Traditional community ecology has focused on local scale mechanisms when trying to explain patterns of species' distributions and abundances. With the development of the metacommunity concept, however, the importance of larger scale mechanisms in structuring diversity has been acknowledged [1], [2]. Connectedness of a set of local communities to a metacommunity can have both positive and negative effects on diversity, depending on the level of connectivity and the spatial scale at which diversity is considered. Connectivity allows immigration of dispersal-limited species and reimmigration of inferior competitors from source communities, thus increasing local diversity [3], [4], [5]. A high degree of connectivity, however, is predicted to result in homogenization of the metacommunity and create one single large community. Here, the regionally superior competitor eliminates inferior competitors from the metacommunity, thus decreasing both local and regional diversity [3], [4]. While empirical studies confirm the positive effect of dispersal on local diversity, experiments do not necessarily find a decline of local and regional diversity with high connectivity [5].

Predation has been revealed as a factor strongly influencing the dispersal-diversity relationship. With an efficient generalist predator present in the metacommunity, the positive effect of connectivity on local richness has been found to be strongly dampened [6], [7]. When the predator eliminates superior competitors, however, connectivity to a regional species pool enables immigration of inferior competitors and an increase in local richness compared to communities without dispersal [8]. Inconsistent results have been detected for the relationship between connectivity and regional richness, with negative, positive, or nonsignificant relationships found in metacommunity experiments with generalist predators [6], [7], [9]. Size-selective predation, however, produced a unimodal relationship between dispersal and regional richness of inedible prey, with no effect found for edible prey [10]. Hence, predator selectivity seems to be an important factor in determining how predator presence influences the relationship between diversity and connectivity.

Apart from predator selectivity, previous metacommunity experiments differed in the way they manipulated predator dispersal. Some studies used similar-sized prey and predators such that connectivity enabled dispersal of both predator and prey [6], [9], while other experiments used predators considerably larger than the prey and dispersal treatments manipulated only prey dispersal [8], [10]. However, whether corridors enable spread of an efficient predator or are connections only for the prey could crucially influence the effect of predation on the dispersal-diversity relationship and might be a reason for differing results of previous experiments.

Here we present an experiment that addresses the effect of connectivity on diversity in metacommunities that were either connected only for the prey or connected for both predator and prey. Both scenarios are also found in natural systems. Spatially patchy habitats like ponds or lakes are connected for phyto- and zooplankton through passive dispersal [11], [12], [13], while planktivorous fish are unable to migrate between ponds without direct connections. This dispersal limitation results in a spatially heterogeneous distribution of the predator and thus regional coexistence of predation-tolerant and predation-susceptible prey species [14], [15]. In habitats without such distinct barriers to dispersal, however, it is often the predators that move over larger spatial scales than the prey [16].

The aims of our study were to analyse the effects of connectivity and predator dispersal on prey species richness, and on prey and predator abundances. In a microcosm experiment, we compared metacommunities that were either unconnected, connected for the prey, or connected for both predator and prey. Four communities with ciliate prey were combined to a metacommunity according to one of three connectivity levels (unconnected, low, or high connectivity). One of the four local communities was stocked with a population of a predatory copepod, an efficient generalist predator. In the low and high connectivity treatments, dispersal corridors were either open for both prey and predator, or blocked for the predator but open for the prey. We hypothesized that the effect of connectivity on prey diversity would strongly depend on whether connectivity enabled only prey dispersal or dispersal of both prey and predator. The positive effect of connectivity on local prey diversity in metacommunities without predator dispersal should turn negative in metacommunities with predator dispersal. The negative effect of connectivity on regional prey diversity should be amplified by predator dispersal. The speed of diversity decline should depend on the level of connectivity, with slow predator dispersal in the low connectivity treatment resulting in a delayed decline in prey species richness. To elucidate possible reasons for species-specific responses to predator dispersal, we measured mortality rates of the prey species and feeding preferences of the copepod in short-term grazing experiments. We further hypothesized that in metacommunities without predator dispersal, reimmigration of prey from predator-free refuges into sink habitats with predators present would maintain higher prey abundances than in isolated patches with predators present. Alternatively, reimmigrating prey could be converted into increased predator abundance.

## Methods

### Ethics statement

No specific permits were required for the described study. The organisms used for the experiments were isolated from locations that are open to the public and are not protected in any way. The study did not involve endangered or protected species.

### Metacommunity experiment

Local communities were small plexiglass basins (12×12×8 cm) filled with 300 ml of 0.2 µm-filtered pond water. To simulate a benthic system, we used small ceramic tiles (2.27×2.27×0.5 cm) as artificial substrate. Four days prior to the experiment, we incubated the tiles with bacillariophycean medium and an inoculum of the benthic diatom *Navicula pelliculosa*, obtained from the culture collection at Göttingen (SAG). At the beginning of the experiment, we placed 25 tiles covered with an algal biofilm into each basin. Since the algal culture was non-axenic, the resource biofilm on the tiles also included a variety of bacteria. Further details on the microcosms can be found in Limberger and Wickham [17].

**Figure 1:**
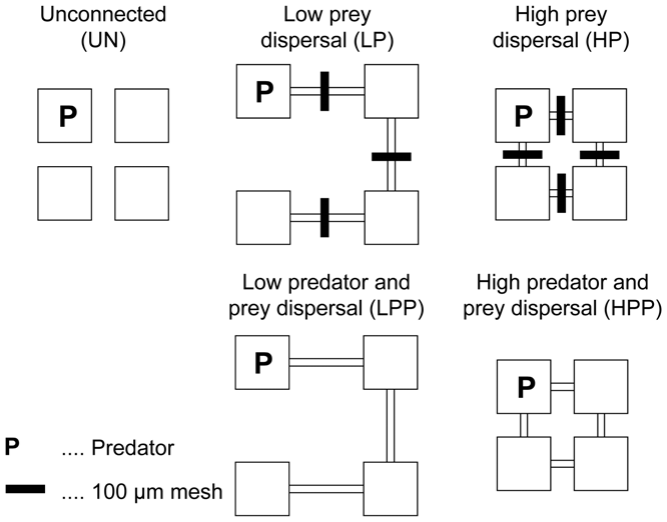
Experimental design of the metacommunity experiment and initial distribution of the predator. Four microcosms were connected according to one of three connectivity levels. Microcosms either remained unconnected (UN), were connected to an open square by tubing of 15 cm length (LP, LPP), or were connected to a closed square by tubing of 5 cm length (HP, HPP). Predatory copepods were introduced into one of the four microcosms on day 7 of the experiment. Copepods were either free to disperse to neighbouring basins (LPP, HPP) or prevented from dispersing by a 100 µm mesh in the middle of the connections (LP, HP).

A metacommunity consisted of four basins, either unconnected or connected by silicon tubing of 0.5 cm inner diameter. We used different lengths and numbers of connections to compare three levels of connectivity: an unconnected control, a low connectivity treatment (1.5 connections per basin, tubing of 15 cm length), and a high connectivity treatment (2 connections per basin, tubing of 5 cm length). In the high connectivity treatment the four basins were arranged in a closed square and each basin was connected with its two neighbouring basins, while in the low connectivity treatment the four basins were arranged in an open square with two basins having only one connection (Fig. 1). We cross-classified low and high connectivity with the second main factor: predator dispersal. In treatments without predator dispersal, we blocked the tubing in the middle by a mesh of 100 µm mesh size. A small piece of a pipette tip (0.5×0.5 cm) that tightly fit into the tubing served to push the mesh into the middle of the corridor and hold it in position. A preliminary experiment showed that all five ciliate species were able to move through the 100 µm mesh. All five species had diameters smaller than 100 µm, and experience has shown that ciliates pass easily even through mesh sizes considerably smaller than their (flexible) diameter. While the mesh allowed prey dispersal, it was efficient in preventing dispersal of the copepod. Only once over the 8-week course of the experiment did two individuals move through a connection, probably when in the stage of nauplii. However, this abundance was too low to have any effect on the prey community of the neighbouring basin. We compared the treatments without predator dispersal to metacommunities without mesh where both prey and predator were able to disperse. All treatments were replicated three times and conducted at 20°C with a light∶day cycle of 12∶12 hours.

The copepod *Diacyclops bicuspidatus* served as predator and five benthic ciliate species (*Tachysoma pellionellum*, *Stylonychia pustulata*, *Frontonia angusta*, *Frontonia atra*, *Paramecium caudatum*) as its prey. We had isolated copepods and ciliates from freshwater habitats around the city of Salzburg, Austria. Data on growth and dispersal rates, colonization and competitive abilities is available for four of the five ciliate species [17]. At the beginning of the experiment, we added 250 individuals of each of the five ciliate species to each basin. One week later, we added 25 individuals of the predatory copepod to one of the four basins. In the low connectivity treatment where local communities differed in the number of connections, we introduced the copepods into one of the end basins with only one connection (Fig. 1). We filtered part of the copepod culture through a 5 µm filter and added the filtrate to the copepod-free basins to ensure that the bacterial community was the same for all local communities. As a control for the effect of ciliate grazing on the algal and bacterial resource community, two additional basins were left without ciliates and copepods but received a filtrate (5 µm) of the ciliate cultures at the start of the experiment and a filtrate of the copepod culture one week later to introduce the same bacterial community.

### Sampling

The experiment lasted for 8 weeks and was sampled once a week for abundances of ciliates, copepods, and resources (algae and bacteria). After blocking the connections with tube clamps, we removed three tiles from each basin with a plexiglass sampler (inner dimensions: 2.27×2.27×9.5 cm). One tile tightly fit into the sampler, allowing removal of the tile and the water column above it. The tiles were replaced by three resource-covered tiles and the removed water was replaced by filtered pond water enriched with nutrients to ensure algal growth. We scraped off the biofilm on the sampled tiles with a razor blade and merged it with the withdrawn water to give a 75 ml sample volume. Ciliates were live-counted under a dissecting microscope. Depending on species' abundances, we counted up to 7.5 ml of the sample. Copepods were counted in the entire sample volume and returned to their respective basin. However, this gave only a rough estimation of copepod abundances. At the end of the experiment, we filtered the whole content of each basin through a 30 µm mesh and live-counted the copepods to differentiate between living and dead individuals. We then fixed living individuals with formaldehyde and measured the length of all or at least 20 individuals per sample. Copepod abundances were converted into dry weight following Dumont et al. [18].

We also used weekly samples to quantify resources. Algal abundances were measured fluorometrically. Fluorescence values were transformed to abundance values after calibrating the fluorometer with samples of known algal concentration. Bacteria were quantified after staining with DAPI. Therefore, a subsample was fixed with glutardialdehyde (2% final concentration) and sonicated to disaggregate clumps of algae and bacteria. A DAPI-stained subsample was then filtered onto black polycarbonate membrane filters (0.2 µm pore size). Bacteria were classified into different size classes (<1 µm, 1–5 µm, 5–10 µm, >10 µm) and counted by epifluorescence microscopy in 30 randomly selected fields. Dimensions of at least 10 individuals per size category were measured to transform counts into biovolume.

### Mortality rates and predator's selectivity

We determined mortality rates for each of the five ciliate species in short-term feeding experiments with single prey species. Ciliate growth was compared in treatments with and without copepods, respectively. We filled beakers of 100 ml volume with 49 ml of a ciliate culture and 1 ml of a diatom culture as resource for the prey. Part of the original culture was fixed with Bouin's solution (5% final concentration) for determination of initial ciliate density. In treatments with predation, we added five individuals of the copepod. After 48 hours, cultures were fixed with Bouin's solution and subsamples were counted under an inverted microscope.

To determine whether the copepod was selectively feeding on some of the prey species, we measured mortality rates of the ciliates in a mixed prey experiment. Cultures of all five prey species were combined and 49 ml of the mixed culture were fed with a 1 ml diatom suspension. Again, ciliate growth was compared in treatments with and without predation. After 24 hours, abundances of the five prey species were determined and used for calculation of prey species' mortality rates and predator's feeding preferences. In contrast to the single prey species feeding experiment, samples from the mixed community experiment were live-counted under a dissecting microscope, since some of the ciliate species were difficult to distinguish when fixed. In both feeding experiments, treatments were replicated five times.

### Data analysis

In the metacommunity experiment, we computed species richness at three spatial scales. Local species richness was the average species number in the four local communities of a metacommunity, regional species richness was the species number in a metacommunity, and beta richness was the difference between regional and average local richness [19]. We used one-way repeated measures (rm) ANOVAs and Tukey's post-hoc tests to test whether the five treatments significantly affected local, regional, and beta richness and abundances of the five ciliate species. Richness and abundance values from days 14 to 56 were used for analyses. When the assumption of sphericity was violated, a Greenhouse-Geisser correction was used. Since the unconnected control was not part of the factorial design but yielded significant differences from other treatments at least for some of the measured parameters, one-way rm-ANOVAs were computed instead of two-way rm-ANOVAs.

To test whether resources differed between treatments, total bacterial biovolume was compared between the five metacommunity treatments and the treatment without animals using one-way rm-ANOVA. A rm-ANCOVA with light as a covariable was conducted to test for treatment effects on algal biovolume. Light intensity was not completely homogeneous throughout the laboratory, and was therefore measured at the position of each basin after the experiment to partial out a possible light effect. Abundances and biovolumina of resources, ciliates, and copepods were log_10_-transformed prior to analyses.

In the feeding experiments, we calculated mortality rate (m; day^−1^) as m = ln(N_−_/N_+_)/t where N_−_ is the final ciliate abundance without predation, N_+_ is the final ciliate abundance with predation and t is the duration of the experiment. To determine feeding preferences of the predator, we calculated Chesson's [20] selectivity index for the mixed prey experiment. For each prey species, selectivity (α) was computed as the ratio of the predator's clearance rate for that prey species and the sum over all clearance rates. Clearance rate (c; ml day^−1^) was calculated as c = m×volume. The selectivity index ranges from 0 to 1 and depends on the number of available prey items. In the case of five prey species, an α>0.2 means selective feeding of the predator on the respective prey species. We used a bootstrap procedure with 10,000 simulations to calculate confidence intervals for mortality rates and the selectivity index. In each of the 10,000 draws, one of the five replicates with and without predation, respectively, was drawn randomly and mortality and selectivity were calculated. Bootstrap analyses were calculated with R 2.10.0 [21], all other analyses were computed with PASW 18.0 for Windows.

## Results

### Species richness

**Figure 2:**
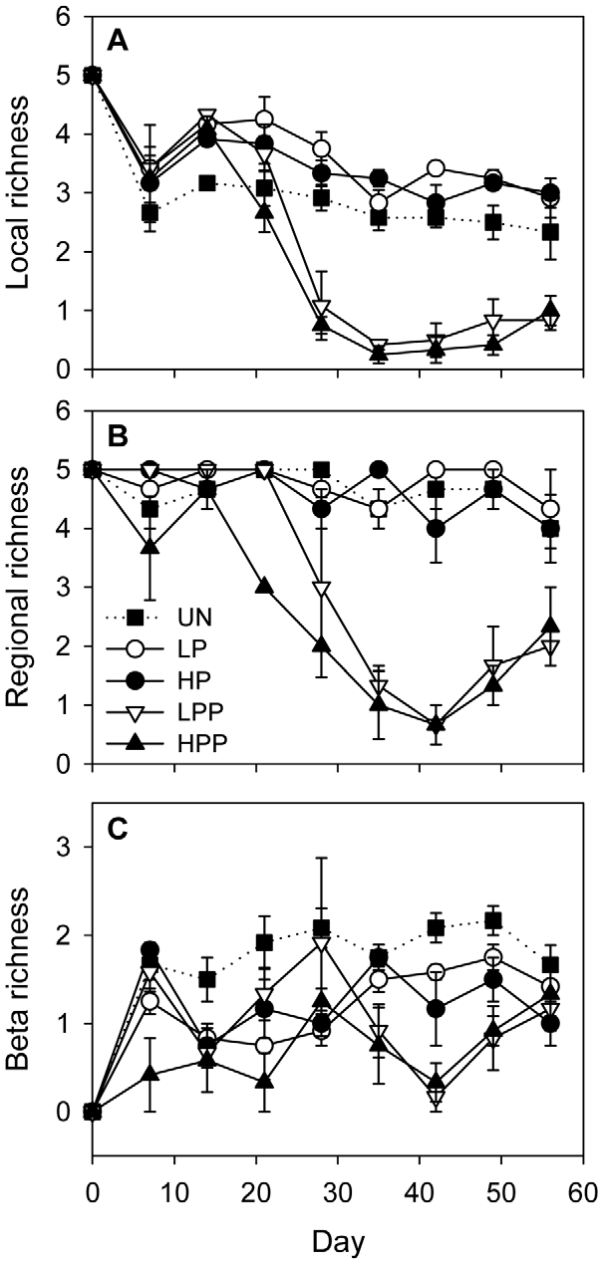
Local, regional, and beta richness of the prey community over the course of the experiment. The experiment compared an unconnected control (UN) and metacommunities with low prey dispersal (LP), high prey dispersal (HP), low predator and prey dispersal (LPP) and high predator and prey dispersal (HPP). Low connectivity is shown with open symbols, high connectivity with closed symboles. Circles mark the treatments with prey dispersal only, and triangles are the treatments with predator and prey dispersal. The unconnected control is shown with dotted line and filled squares. Values are means ± SE (n = 3).

The generalist predator had a strong negative effect on prey species richness, reducing it to low levels in those basins to which it had access (final species number in basins with predator access: 0.76, SE = 0.12, n = 33; and in basins without predator access: 3.56, SE = 0.17, n = 27). Thus, local species richness was significantly reduced when the predator was able to disperse over the metacommunity (Table 1, Fig. 2A). However, the effect of treatment strongly interacted with time. In the beginning, local species richness was lowest in the unconnected control (one-way ANOVA day 14: P = 0.001; Tukey: UN<all other treatments; treatment abbreviation as in Fig. 2), while the negative effect of predator dispersal became apparent on day 21 (high predator dispersal) and day 28 (low predator dispersal), respectively. At the metacommunity scale, richness was also strongly reduced in those treatments that allowed predator dispersal (Table 1, Fig. 2B). Again, the speed in richness decline depended on the level of connectivity. After three weeks, regional richness had been reduced to three species in the treatment with high predator dispersal, while all five species still were present in the metacommunities with low predator dispersal. On day 28, metacommunity richness was highly variable with low predator dispersal: while all five species had gone extinct in one of the replicates, four and five species, respectively, still survived in the other two replicates. During the second half of the experiment, predator dispersal reduced regional richness to low values irrespective of the level of connectivity.

**Table 1:** P-values and results of Tukey's post-hoc tests of one-way rm-ANOVAs testing for treatment effects on local, regional, and beta richness and on the abundances of the five ciliate species.

	time	time×treatment	treatment	Tukey's post-hoc test
local richness	**<0.001**	**<0.001**	**<0.001**	HPP, LPP<HP, LP, UN
regional richness	**<0.001**	**<0.001**	**<0.001**	HPP, LPP<HP, LP, UN
beta richness	**0.042**	0.113	**<0.001**	HPP, LPP, HP, LP<UN and HPP<LP
*Tachysoma*	**<0.001**	**0.001**	**<0.001**	HPP, LPP<HP, LP, UN
*Stylonychia*	**<0.001**	**<0.001**	**<0.001**	HPP, LPP<HP, LP, UN
*F. angusta*	**<0.001**	0.198	0.336	
*F. atra*	**<0.001**	**0.001**	**0.001**	HPP<HP, LP, UN and LPP<HP
*Paramecium*	**<0.001**	**<0.001**	**<0.001**	HPP<HP, LP, UN and LPP<HP, LP

UN = unconnected, LP = low prey dispersal, HP = high prey dispersal, LPP = low predator and prey dispersal, HPP = high predator and prey dispersal; P-values<0.05 are bold; n = 3.

Beta richness was consistently highest in the unconnected control (Table 1, Fig. 2C), while being comparatively low in metacommunities with high predator dispersal. With low predator dispersal, however, beta richness was highly variable with time: it was high on days 21 and 28 and then declined to values similarly low as with high predator dispersal.

### Species' abundances

**Figure 3:**
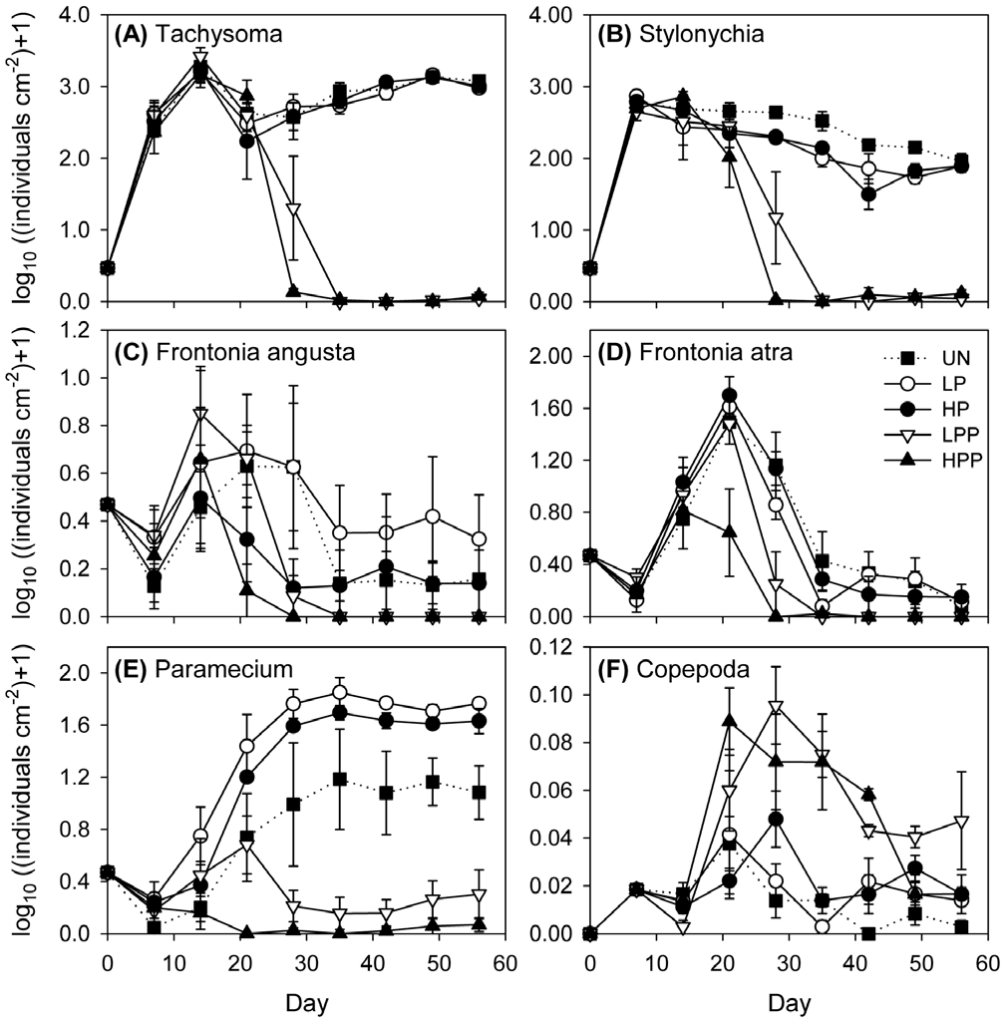
Metacommunity abundances of all prey species and of the predatory copepod. Treatment abbreviations and symbols as in Fig. 2. Abundances (log_10_ transformed) are means ± SE (n = 3).

All five prey species were driven to extinction or low abundances by the predatory copepod. Four of them (*Tachysoma*, *Stylonychia*, *Frontonia atra*, *Paramecium*) showed a strong negative response to the treatments with predator dispersal (Table 1, Fig. 3). The fifth species, *Frontonia angusta*, reached only low abundances in all treatments and showed large variation between basins and replicates (Fig. 3C). Thus, no statistically significant effects were detected for this species.

While an effect of connectivity on species' abundances was not apparent when averaging over time, the speed of abundance decline during the middle period of the experiment depended on the level of connectivity (Fig. 3). The four species with significant responses to predator dispersal were driven to extinction or to low abundances one week earlier in treatments with high predator dispersal than in treatments with low predator dispersal. For *Paramecium*, the level of connectivity also played a role in the three treatments without predator dispersal (Fig. 3E). *Paramecium* went extinct in some basins of the unconnected control treatment and thus on average reached lower abundances in metacommunities without connections than when the basins were connected for the prey. Due to high variability between replicates, however, this difference was not statistically significant.

Time to extinction in metacommunities with predator dispersal not only depended on the level of connectivity, it also differed between the ciliate species. *Tachysoma* and *Stylonychia*, the two smallest and fastest-growing species, responded very similarly to the treatments: high predator dispersal drove them to low abundances or extinction on day 28, while with low predator dispersal extinction was delayed for one week, at least in two of the three replicates (Table 1, Fig. 3A, B). Compared to *Tachysoma* and *Stylonychia*, abundances of *F. atra* and *Paramecium* were affected by high predator dispersal one and two weeks earlier, respectively.

To test whether ciliates were able to maintain higher populations through reimmigration from spatial refuges, total ciliate abundance in the basin with the predator was compared between the unconnected treatment and the two treatments with prey dispersal only. However, total prey abundances were reduced to low or zero abundances irrespective of the level of prey connectivity (rm-ANOVA: time: P<0.001, time×treatment: P = 0.750, treatment: P = 0.616).

**Figure 4:**
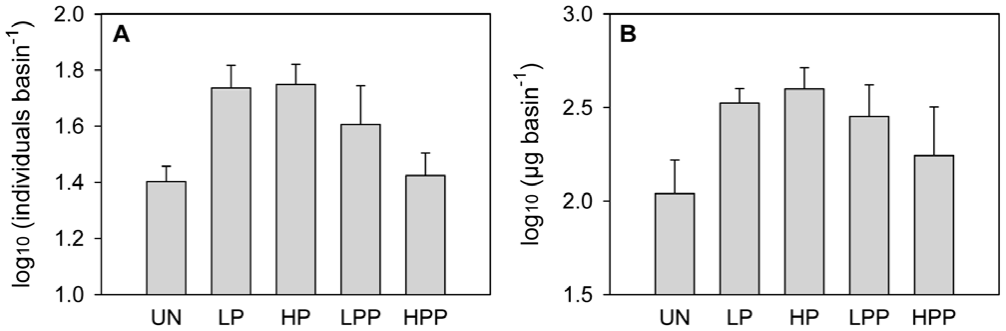
Final copepod abundance (A) and dry weight (B). Only those basins to which the predator had access were considered for calculations. Values are means ± SE (n = 3).

Counts of copepods during the experiment have to be regarded with caution but are shown here to give a rough picture of the time course of copepod abundances (Fig. 3F). Regional copepod abundances were higher in the treatments with predator dispersal since the predator had access to a larger habitat. It increased in abundance and remained high from days 21 to 42, concurrent with the decline in prey abundance, and then decreased when the prey had been depleted. For a comparison of treatment effects on final copepod abundance and dry weight, values were averaged only over those local communities to which the copepods had access. In the end of the experiment, copepod abundances nearly significantly differed between treatments (one-way ANOVA: P = 0.053, Fig. 4A), with the unconnected control containing fewer copepods than metacommunities connected only for the prey. When transformed to dry weight, however, no significant treatment effects were found (one-way ANOVA: P = 0.213, Fig. 4B).

### Resources

**Figure 5:**
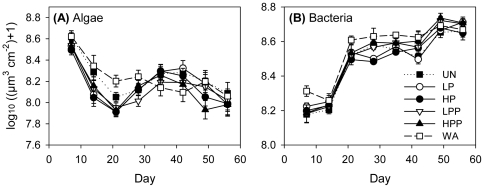
Algal biovolume (A) and total bacterial biovolume (B) in the metacommunities. Treatment abbreviations and symbols as in Fig. 2. Additionally, resources were measured in isolated basins without animals (WA), shown with dashed line and open squares. Values are means ± SE (n = 2 for WA, n = 3 for all other treatments).

Algal biovolume did not differ between treatments after accounting for the effect of light (rm-ANCOVA: time: P = 0.443, time×light: P = 0.699, time×treatment: P = 0.286, light: P<0.001, treatment: P = 0.402, Fig. 5A). Total bacterial biovolume did not differ between the five predator treatments but was comparatively high in the basins without any animals (rm-ANOVA: time: P<0.001, time×treatment: P = 0.267, treatment: P = 0.035; Tukey's post-hoc test: HP<WA, Fig. 5B).

### Mortality rates and selectivity

Mortality rates of several species considerably differed between the single species trials and the experiment with a mixed prey community (Table 2). The two smallest species had much lower mortality rates in the mixed prey community than when offered to the predator as the only prey. *Paramecium*, however, had fewer losses to predation in the single species trial than in the mixed prey experiment.

**Table 2:** Ciliate biovolume (10^3^ µm^3^; n = 15), mortality rate (day^−1^) in the single prey species experiment, and mortality rate and selectivity in the mixed prey species experiment.

Species	Biovolume	Mortality rate Single species trial	Mortality rate Mixed community	Selectivity
*Tachysoma*	17 (1.1)	0.51 (0.08; 0.73)	−0.22 (−0.54; 0.23)	−0.07 (−0.23; 0.1)
*Stylonychia*	31 (1.8)	1.79 (1.31; 2.2)	0.47 (0.1; 0.91)	0.18 (0.02; 0.43)
*F. angusta*	89 (5.9)	0.93 (−0.04; 1.95)	0.67 (0.17; 1.72)	0.22 (0.06; 0.37)
*F. atra*	208 (11.1)	0.74 (0.04; 1.42)	0.67 (0.1; 1.22)	0.24 (0.05; 0.45)
*Paramecium*	216 (20.9)	0.73 (0.11; 1.14)	1.54 (0; 3.99)	0.44 (0; 0.68)

Ciliate biovolume with standard error in brackets. Mortality rates and selectivity were averaged over 10,000 random draws from 5 replicates, 2.5 and 97.5 percentiles in brackets.

The prey species differed in their susceptibility to predation: after 24 h of grazing on the mixed prey community, *Paramecium* was depleted the strongest. However, the confidence intervals for its mortality rate and for the selectivity index were rather large since in one of the possible comparisons of replicates with and without predation abundances of *Paramecium* were the same and mortality was thus 0. *Tachysoma* was least affected by predation, its mortality rate in the mixed prey community not differing significantly from 0. Its abundances were higher in some of the replicates with than without predators, resulting in a negative mortality rate.

## Discussion

### Prey species richness

The effect of connectivity on diversity strongly depended on whether corridors were open only for the prey species or for both prey and predator (Fig. 2). Without predator dispersal, local richness was slightly higher in connected than in unconnected communities, while connectivity had no effect on regional richness. When communities were connected for both predator and prey, local and regional richness were both strongly reduced compared to the unconnected control.

The mechanism behind the positive effect of prey dispersal on local richness, apparent especially during the initial phase of the experiment, was the rescue of species from stochastic extinctions. Although all ciliate species had been introduced with the same initial abundances, the three large and slow-growing species went extinct in many of the isolated communities, also in the absence of the predator. *Paramecium* was especially prone to stochastic extinctions and was found in only four of the nine isolated, predator-free basins. In connected metacommunities, however, such populations were rescued by reimmigration from neighbouring communities. While we did find, at least temporally, an increase in local diversity in connected relative to unconnected communities similar to other metacommunity experiments [5], we did not find a decline of local richness with high connectivity predicted by modeling approaches [3], [4]. Due to identical prey species composition in all local communities at the beginning of our experiment, the competitively dominant species (*Tachysoma*, *Stylonychia*) were present in every local community. Moreover, they were the species with the highest growth rates and hence not prone to stochastic extinctions. There was thus no reason why high connectivity should lead to a further increase in their dominance.

When connections enabled dispersal of both prey and predator, the initially heterogeneously distributed predator dispersed over the entire metacommunity, negatively affecting both local and regional richness. Similarly, Cadotte and Fukami [9] found that dispersal of a generalist predator resulted in removal of predator-free refuges, thus decreasing regional richness and negating the initially positive effect of connectivity on local richness. The speed of richness decline in our treatments with predator dispersal depended on the level of connectivity. This effect was particularly pronounced for regional richness which was significantly less with high than with low connectivity on day 21 of the experiment. High predator dispersal resulted in quick homogenization of the predator's distribution, fast prey depletion in all local communities and thus low regional richness within a few weeks. With low predator dispersal, prey was soon depleted in the predator's source community, but all or most species still survived in communities farther away, maintaining high regional richness longer than with high predator dispersal (Fig. 2B). Low connectivity thus created a spatial mosaic of patches with differing predation pressure, resulting in temporally high beta richness (Fig. 2C). However, after dispersal of the predator over the metacommunity and increase in its population size in all local communities, the predator also caused regional prey species extinctions in the weakly connected treatment. Thus, among the two antagonistic effects of connectivity, rescue from stochastic extinctions but increased deterministic extinctions through spread of strong competitors and predators [22], it was clearly the negative effect that outweighed the positive when connectivity enabled dispersal of the predator.

While regional richness was negatively affected by connectivity when it allowed dispersal of the predator, regional richness was unaffected by connectivity when corridors were open only for the prey species. These results may give a hint on why the predicted decline of regional richness with connectivity [4] is not found by all empirical studies on this subject [5]. Our results, in comparison with those of previous metacommunity experiments, suggest three prerequisites for a negative effect of connectivity on regional richness: First, a strong negative interaction (e.g. with a strong competitor or an efficient generalist predator) has to be present in the metacommunity. Second, this negative interaction has to be initially heterogeneously distributed over the metacommunity. Only then can connectivity lead to homogenization, whereas isolation maintains spatial refuges from the negative interaction. And third, this negative interaction has to be able to disperse through the corridors. Only when this strong competitor or predator is able to disperse over the entire metacommunity and drives the same species extinct in every single local community, will regional richness be lower in highly connected than in isolated or weakly connected communities.

Accordingly, previous metacommunity experiments without initial variation in the distribution of competitors or predators found no effect of dispersal on regional richness [6], [23], while regional richness declined with dispersal in experiments where local communities differed in initial community composition [9], [23] or in environmental conditions [24]. Spatial heterogeneity alone, however, is not sufficient for a negative effect of connectivity on regional richness. Rather, species interactions need to be strong enough to cause regional extinctions. In a metacommunity experiment with benthic microalgae, regional richness was unaffected by dispersal despite initial variation in local community composition [25]. Declining beta-diversity with increasing dispersal frequency indicated homogenization of the metacommunity, but competitive interactions were apparently not strong enough to exclude the same species from every local community. Likewise, metacommunity experiments with spatial heterogeneity in environmental conditions do not necessarily find an effect of connectivity on regional richness [26], [27].

In addition to the presence and heterogeneous distribution of a strong negative interaction, its dispersal is a further prerequisite for a negative effect of connectivity on regional richness. Despite spatial heterogeneity in the distribution of a predator, Howeth and Leibold [10] found similar regional prey species richness in unconnected and highly connected metacommunities. However, local communities were connected only for the prey, while the predator was unable to disperse and thus predator-free refuges remained despite high connectivity. Similarly, regional richness did not decrease with connectivity in our treatments without predator dispersal, as regional extinctions were prevented through survival of prey species in spatial refuges. Competitive interactions among the prey species were weak and resulted only in few regional extinctions. Hence, only when corridors were open for the predator did connectivity result in a decline of regional richness, through homogenization of the predator's distribution and removal of the predator-free refuges.

Apart from negative and nonsignificant relationships between regional richness and connectivity, some experiments found an increase in regional richness in connected relative to unconnected metacommunities [7], [28]. Here, isolation resulted in reduced species' abundances and thus higher extinction probabilities of species with small population sizes. Large predators with small densities were particularly extinction-prone in small isolated patches [28], [29]. While there also were stochastic extinctions in our unconnected metacommunities, none of the prey species was so extinction-prone that stochastic extinctions eliminated it from the whole landscape. Stochastic extinctions in our metacommunity thus affected only average local richness, while it took strong deterministic extinctions by a predator to cause regional extinctions.

### Species-specific responses to predation

All of the prey species were driven to extinction or to very low abundances by the predatory copepod, with species-specific differences in the speed of decline (Fig. 3). Large prey species (*F. atra*, *Paramecium*) were affected earlier than the two smallest and fastest-growing species (*Tachysoma*, *Stylonychia*). However, the time to regional extinction in treatments with predator dispersal differed not only between the species, it also depended on the level of connectivity. Prey species survived longer in the metacommunity when connectivity was weak and the predator thus spread only slowly over the metacommunity.

Reasons for the species-specific speed of decline in response to predation can be elucidated with the help of the short-term predation experiments (Table 2). Time to exclusion through predation is determined by the prey's vulnerability to predation, its growth rate, and the predator's abundance [30]. High susceptibility to predation and a comparatively low growth rate explain *Paramecium's* fast extinction in treatments with predator dispersal. Slow growth and low relative abundance also resulted in *F. atra* being quickly affected by predation, especially with high predator dispersal. In contrast, the two species that were the last to be affected by predation had high growth rates [17], thus quickly reaching high abundances (Fig. 3A, B) and dominating the early-successional stage of the community. With only mild predation pressure during the initial phase of the experiment, *Tachysoma* and *Stylonychia* were probably able to compensate losses due to predation by fast growth and may have even profited from reduced competitive interactions. With increasing predator abundance, however, these two species also were eliminated by predation. In addition to high growth rates, comparatively low vulnerability to predation probably delayed their extinction. Results for species-specific responses to predation are in accordance with other empirical studies that found common prey species to be less prone to extinction by a generalist predator than rare prey species [31], [32].

It is possible, though unlikely, that the presence of the mesh affected the ciliate community other than via preventing dispersal of the predator. While a preliminary experiment showed that all five ciliate species were able to move through the 100 µm mesh, we cannot exclude that the presence of the mesh reduced the ciliates' dispersal rates. However, all five prey species had diameters considerably smaller than the 100 µm mesh size, ranging from 25 µm for *Tachysoma* to 75 µm for *F. atra*. Moreover, during the initial phase of the experiment, when predator dispersal had not yet affected the ciliate community, diversity and ciliate abundances were highly similar in connected metacommunities both with and without mesh (Fig. 2 and 3), suggesting that a possible reduction in ciliate dispersal rates by the mesh did not affect the results.

### Connectivity and predator abundance

We hypothesized that reimmigration of prey from predator-free refuges into basins with predators would maintain higher prey abundances in these sink habitats compared to the respective basins in unconnected metacommunities. This was not the case, however, since reimmigrating prey was apparently directly converted into increased predator abundance (Fig. 4). Similarly, immigration of zooplankton into local communities with planktivorous fish has been found to result in higher growth rates of the predator compared to isolated communities [33]. Hence, connectivity can have a positive effect on a predator, even if the predator itself does not disperse to prey refuges, but connections enable immigration of prey. With even stronger connectivity than tested in our experiment, still higher predator abundances might have been sustained through high prey dispersal.

Habitats that are sinks through presence of predators may thus have a different effect than habitats that are sinks through environmental conditions. Source-sink metacommunity experiments with local habitats differing in resource quality or quantity found species' abundances and richness in sink habitats to be higher when sinks were connected to source habitats [26], [34]. Prey dispersal to sink habitats with predators, however, just leads to increased predation pressure through conversion of reimmigrating prey into higher predator abundances, rather than sustaining prey populations.

### Conclusions

Dispersal of a generalist predator strongly influenced the relationship between connectivity and diversity. The positive effect of connectivity on local richness soon turned negative when the predator was able to disperse over the metacommunity. The positive dispersal effect through rescue from stochastic extinctions was then outweighed by extinctions through predation. While regional richness was unaffected by connectivity when only prey species were able to disperse, predator dispersal resulted in a negative effect of connectivity on regional richness. These results suggest that it is dispersal of an initially heterogeneously distributed species with strong negative effects on other community members that is a prerequisite for the predicted decline of regional richness with connectivity.
